# Does Luteal Phase Support Effect Pregnancy Rates in Intrauterine Insemination Cycles? A Prospective Randomised Controlled Study in a Tertiary Center

**DOI:** 10.1155/2020/6234070

**Published:** 2020-08-05

**Authors:** Müge Keskin, Ruşen Aytaç

**Affiliations:** ^1^Department of Obstetrics and Gynecology, Ufuk University Faculty of Medicine, Ankara, Turkey; ^2^Department of Obstetrics and Gynecology, Ankara University Faculty of Medicine, Ankara, Turkey

## Abstract

Intrauterine insemination (IUI) is a common treatment for couples with subfertility. Clomiphene citrate, gonadotropins, and letrozole are used for ovulation induction in IUI cycles. It has been well documented that luteal support with exogenous progesterone after in vitro fertilization is associated with higher pregnancy and live birth rates. Yet, luteal phase support in IUI cycles has become a debatable issue. The aim of this prospective controlled study was to assess the effect of luteal phase vaginal progesterone supplementation on *β*-hCG positivity and clinical pregnancy rates in women undergoing IUI. This prospective controlled randomised study was conducted at a tertiary infertility center. 87 patients with unexplained infertility or male subfertility who were treated with IUI using gonadotropins were enrolled. Patients in the study group (*n* = 44) received luteal phase vaginal progesterone supplementation. Patients in the control group (*n* = 43) did not receive any luteal phase support. There was no statistical difference between two groups in terms of *β*-hCG positivity and clinical pregnancy rates. Our findings do not show any beneficial effect of luteal phase support in IUI cycles stimulated with gonadotropins. Although luteal phase support in IUI cycles stimulated with gonadotropins is widely adopted, there is a lack of robust evidence.

## 1. Introduction

Intrauterine insemination (IUI) is a common treatment for couples with subfertility with lower costs compared to in vitro fertilization (IVF) [1, 2]. Clomiphene citrate, gonadotropins, and letrozole are used for ovulation induction in IUI cycles [3]. Patients undergo controlled ovarian stimulation with these agents before the procedure in an attempt to increase the number of oocytes and eliminate ovulation disorders [4].

Luteal phase is defined as the period between ovulation and the end of the menstrual cycle marked by either onset of the menstruation or onset of pregnancy [5]. Following ovulation, the luteal phase of a natural cycle is characterized by the formation of a corpus luteum (CL) which secretes steroid hormones including progesterone (P) and estradiol (E2). If conception and implantation occurs, the developing blastocyst secretes human chorionic gonadotropin (hCG) for the maintenance of CL and its secretions [6]. P is required for endometrial receptivity and its secretory transformation [7].

Fertility treatments may interfere with the luteal phase via several mechanisms. Disruptions in the hypothalamic-pituitary-gonadal axis as a consequence of supraphysiologic E2 levels caused by controlled ovarian stimulation lead to a shortened luteal phase with low concentrations of P [8]. Therefore, in assisted reproductive technology (ART), ovarian stimulation with gonadotropins is associated with luteal phase deficiency which can be compensated with the luteal phase support [9].

It has been well documented that luteal support with exogenous P after ART is associated with higher pregnancy and live birth rates [10, 11].

Supraphysiologic E2 levels are often associated with multifollicular development during assisted reproductive technology (ART) [12]. Yet, during ovulation induction in IUI, only one to two dominant follicles may be achieved which makes the influence of mild ovarian stimulation on the corpus luteum function questionable. As a result, luteal phase support in IUI cycles has become a debatable issue with controversial findings in many different studies.

The aim of this prospective controlled study was to assess the effect of luteal phase vaginal progesterone supplementation on *β*-hCG positivity and clinical pregnancy rates in women undergoing ovarian stimulation and IUI.

## 2. Materials and Methods

This prospective controlled randomised study was conducted at the Ankara University School of Medicine Infertility Center, a tertiary infertility center, from August 2014 to January 2015. 87 patients with unexplained infertility or male subfertility who were treated with ovarian stimulation and IUI using gonadotropins were enrolled. The study was approved by the ethics committee of the Ankara University School of Medicine, and all couples gave informed consent before entering the study. Patients were divided into two groups by computer-generated random allocation. Patients in the study group (*n* = 44) received luteal phase vaginal progesterone supplementation. Patients in the control group (*n* = 43) did not receive any luteal phase support.

Inclusion criteria were stated as follows: duration of infertility at least 2 years, age ≤ 35 years, undergoing first IUI cycle, basal (3rd day of the menstrual cycle) FSH level < 12 mIU/mL, normal serum prolactin (PRL) and thyroid-stimulating hormone (TSH) levels, body mass index (BMI) ranging between 18 and 25 kg/m^2^, hysterosalpingography showing a normal uterine cavity and bilateral tubal patency, male subfertility, and unexplained infertility. Male subfertility was defined as a sperm count greater than 5 million/ml and less than 20 million/ml. The exclusion criteria included the previous ART cycle, age >35 years, diminished ovarian reserve (basal FSH level > 12 mIU/mL or antral follicle count<4), hyperprolactinemia, thyroid dysfunction, and other endocrine disorders including adrenal pathologies or diabetes mellitus (DM). Additionally, patients with severe oligozoospermia (sperm count < 5 million/ml) were excluded.

After the evaluation of serum FSH, LH, estradiol, PRL, and TSH levels on the third day of the menstrual cycle, all patients underwent baseline transvaginal ultrasonography for antral follicle count (AFC). Then, controlled ovarian stimulation was performed using menotrophin, follitropin *α*, follitropin *β*, or highly purified human menopausal gonadotropin (hpHMG). Ovarian response was assessed with transvaginal ultrasound (TV-US) from day 8 of each cycle. Cycles were triggered with 10.000 IU hCG when at least one dominant follicle had reached 17 mm in diameter. The IUI was performed 36 h after hCG administration.

In the study group, luteal phase support was provided via vaginal administration of micronized progesterone capsules (Progestan 100 mg, Koçak Farma, Turkey) twice a day beginning on the day of insemination until the monitorizaton of fetal heart rates on TV-US. Patients in the control group did not receive any luteal phase support.

Pregnancy testing was performed by determining the serum hCG level 14 days after hCG administration. Primary outcomes were *β*-hCG positivity and clinical pregnancy rates. *β*-hCG positivity was defined as increased serum hCG levels. Clinical pregnancy was determined as the presence of a gestational sac with embryonic viability on TV-US.

## 3. Statistical Analysis

The Statistical Program for Social Sciences (SPSS, version 11.5; SPSS, Chicago, IL) was used for statistical analysis. Demographic data of the study and control were expressed as mean ± SD. Student's *t*-test was used for normally distributed variables. The Mann–Whitney *U*-test was used for the variables that were not distributed normally. For comparison of pregnancy rates of the patients, the chi-square test was used. The *p* < 0.05 value was accepted statistically significant for all results.

## 4. Results

87 couples either with unexplained infertility or male subfertility were included in the study. Demographic characteristics of the patients are summarised in Table 1. Two groups were comparable in terms of age, BMI, basal FSH, E2, LH, TSH, and PRL levels, duration of infertility, and percentage of patients with unexplained infertility and male subfertility. Basal sperm count was significantly higher in the control group compared to the study group. However, after sperm washing, there was no statistical significance in sperm count and motility between two groups.

Cycle characteristics are also shown in Table 1. There was no difference in duration of stimulation, total amount of gonadotropins, number of follicles with a diameter ≥17 mm, and endometrial thickness on the day of hCG between two groups. Types of gonadotropins used for stimulation were also comparable for follitropin *α*, follitropin *β*, and menotrophin between the two groups. hpHMG use was significantly higher in the control group.

There was no statistical difference between control and study groups in terms of *β*-hCG positivity and clinical pregnancy rates (Table 2).

## 5. Discussion

In this prospective randomised controlled study, it was shown that in patients having ovarian stimulation with gonadotropins for IUI due to unexplained infertility or male subfertility, luteal phase support with vaginal progesterone is not associated with higher *β*-hCG positivity and clinical pregnancy rates compared with patients without luteal phase support.

It has been already proven that all stimulated IVF cycles have luteal phase defect [5] as a result of supraphysiological levels of estradiol secreted by the high number of corpora lutea during the early luteal phase, which directly inhibits LH release via negative feedback actions at the level of the hypothalamic-pituitary axis [13]. Yet, it is a matter of debate whether this fact is true for the IUI cycles where mild ovarian stimulation is applied. Therefore, necessity of luteal phase support in IUI cycles still remains as an unresolved issue.

There are many studies with conflicting results. Maher reported that clinical pregnancy rates and live birth rates were higher in IUI cycles supplemented with vaginal progesterone gel in the luteal phase when rFSH was used [14]. Similarly, Agha-Hosseini et al. showed the use of vaginal suppositories as luteal phase support significantly improved clinical pregnancy rates in controlled ovarian stimulation and intrauterine insemination in patients with unexplained or mild male factor infertility in a prospective randomised controlled study [15]. Erdem et al.'s findings were also in favor of luteal phase support [16]. In contrast, Kyrou et al. concluded that routine supplementation of the luteal phase with vaginal progesterone does not seem to improve pregnancy rates in normoovulatory women stimulated with clomiphene citrate for IUI [17]. In agreement with these findings, Ebrahimi et al. could not show any beneficial effect of luteal phase support with progesterone in IUI cycles stimulated with clomiphene citrate (CC) plus hMG [18].

In a review and meta-analysis including the above mentioned studies, Miralpeix et al. concluded that the supplementation of luteal phase with vaginal progesterone significantly increases live birth among women undergoing IUI when receiving gonadotropins for ovulation induction, and women receiving CC to induce ovulation do not seem to benefit from this treatment [19]. These findings were consistent with two other systematic review and meta-analysis conducted by Green et al. and Hill et al. [20, 21].

On the contrary, our findings are not in favor of luteal phase support as clinical pregnancy rates were comparable between two groups. Nieto et al. reported that in infertile patients treated with mildly ovarian stimulation with recombinant gonadotropins and IUI, luteal phase support with vaginal progesterone is not associated with a higher live birth rate or clinical pregnancy rate compared with patients who did not receive any luteal phase support [22]. Similar to these findings, a recent large multicenter randomised controlled study demonstrated that in patients treated with IUI after ovarian stimulation with gonadotropins, the clinical pregnancy rate was not statistically significantly higher after luteal phase support with a vaginal progesterone gel [23].

In conclusion, it is well proven that stimulated IVF cycles require luteal phase support. Although luteal phase support in IUI cycles stimulated with gonadotropins is widely adopted, there is a lack of robust evidence. Our findings do not show any beneficial effect of luteal phase support in IUI cycles stimulated with gonadotropins. But, our small sample size may be a limitation of our study. Further randomised trials with larger groups are required to examine the necessity of luteal phase support in IUI cycles.

## Figures and Tables

**Table 1:** Patient demographic characteristics and cycle characteristics.

	Control group (*n* = 43)	Study group (*n* = 44)	*p* value
Age			
Median (min-max)	27 (21–34)	28 (22–33)	0.27
Mean ± SD	26.7 ± 3.9	28 ± 4.1	
BMI (kg/m^2^)			
Median (min-max)	23.2(18–25)	22.1(10–24.6)	0.78
Mean ± SD	22.1 ± 2.8	21.6 ± 2.8	
Duration of infertility (yrs) (mean ± SD)	2.8 ± 3	2.6 ± 3.5	0.27
Cause of infertility			
Unexplained	42 (97.6%)	42 (95.4%)	0.074
Male subfertility	1 (2.3%)	2 (4.7%)	
Basal sperm count (×10^6^)			
Mean±SD	105.5 ± 58.2	89.3 ± 61.3	0.042
Median (min-max)	105 (11–320)	83 (12–250)	
Sperm count after washing (mean ± SD)	56.7 ± 31.3	55 ± 29.2	0.41
Sperm motility after washing (%)	51.3% ± 14.7	50.2% ± 15.3	0.47
Basal FSH (mIU/mL)			
Mean	5.9	6.1	0.25
Median (min-max)	7 (3−10.2)	7.1 (3.5–10.1)	
Basal E2 (pg/mL)			
Mean	46.3	47.2	0.77
Median (min-max)	35 (20–60)	37 (10–60)	
Basal progesterone (ng/mL) (mean ± SD)	2.3 ± 3.1	2.5 ± 2.9	0.90
Basal LH (mIU/mL) (mean ± SD)	7.2 ± 2.8	5.6 ± 3.4	0.52
Basal PRL (ng/mL) (mean ± SD)	12 ± 3.4	14.1 ± 7.6	0.43
Basal TSH (*µ*IU/mL) (mean ± SD)	1.9 ± 1.2	2,7 ± 1,6	0.35
Antral follicle count			
Mean±SD	6.9 ± 2.1	7.1 ± 3.4	0.31
Median (min-max)	8 (4−12)	9 (4–13)	
Endometrial thickness on the day of hCG (mm) (mean ± SD)	11.2 ± 1.6	10.9 ± 1.4	0.92
Number of follicles with a diameter of 9–16 mm on the day of hCG			
Mean±SD	3 ± 1.6	2.5 ± 1.5	0.54
Median (min-max)	3 (0–5)	4 (0–6)	
Number of follicles with a diameter ≥17 mm on the day of hCG			
Mean±SD	1.1 ± 0.5	1.4 ± 0.6	0.34
Median (min-max)	2 (1–2)	2 (1–2)	
Gonadotropin type			
Menotrophin	2 (4.6%)	4 (9.09%)	0.81
hpHMG	14 (32.5%)	1 (2.27%)	0.03
Follitropin *α*	9 (20.9%)	10 (22.7%)	0.90
Follitropin *β*	18 (41.8%)	29 (65.9%)	0.08
Duration of stimulation (days) (mean ± SD)	6.7 ± 3.4	7.2 ± 2.4	0.72
Total amount of gonadotropin (IU)	850 ± 510	890 ± 610	0.43

**Table 2:** IUI outcomes.

	Control group (*n* = 43)	Study group (*n* = 44)	*p* value
*β*-hCG positivity	6 (13.9%)	7 (15.9%)	0.76
Clinical pregnancy	6 (13.9%)	3 (6.8%)	0.48

## Data Availability

The data used to support the findings of this study are available from the corresponding author (Keskin M) upon request.
